# Fat Oxidation of Fatty Fish vs. Meat Meal Diets Under *in vitro* Standardized Semi-Dynamic Gastric Digestion

**DOI:** 10.3389/fnut.2022.901006

**Published:** 2022-06-30

**Authors:** Iskandar Azmy Harahap, Maria Madalena C. Sobral, Susana Casal, Susana C. M. Pinho, Miguel A. Faria, Joanna Suliburska, Isabel M. P. L. V. O. Ferreira

**Affiliations:** ^1^Department of Human Nutrition and Dietetics, Faculty of Food Science and Nutrition, Poznan University of Life Sciences, Poznan, Poland; ^2^LAQV/REQUIMTE, Departamento de Ciências Químicas, Laboratório de Bromatologia e Hidrologia, Faculdade de Farmácia, Universidade do Porto, Porto, Portugal

**Keywords:** conjugated dienes and trienes, TBARS, triacylglyceride, lipid oxidation, *in vitro* gastric digestion, fatty acids

## Abstract

Meat and fish are introduced into the diet as a source of protein, but these muscle foods present different fatty acid (FA) compositions and different lipid stabilities. Fatty fish is expected to oxidize due to its higher content of polyunsaturated FA (PUFA), whereas the higher heme-Fe content of red meat will also affect lipid stability. Combining other food ingredients within a meal also influences lipid oxidation, which will not stop after meals intake. This is due to the acidic environment of the stomach together with the presence of metallic ions, a process that is scarcely understood. The goal of this study was to evaluate the oxidation of fatty fish vs. meat meal diets under *in vitro* standardized semi-dynamic gastric conditions and FA release from the stomach to the duodenum. Meal diets composed by 25% beef meal (BM) or fatty fish meal (FM), 25% fried potatoes, and 50% sugar soft drink were prepared. Proximate composition, FA and amino acid profiles, and meals quality indices were evaluated. Their differences in composition led to different total gastric digestion time of 242.74 (BM) and 175.20 (FM) minutes. Using the INFOGEST semi-dynamic gastric model, 4 gastric emptying (GE) were simulated in both meals. In each GE, FA profile and lipid oxidation products (LOPs) formation were assessed. As a result, more than 50% FA release to the duodenum occurred in GE1, whose percentage decreased with the time of digestion. FM exhibited the highest LOPs formation, which corroborates the high peroxidizability index measured for this meal diet. Higher LOPs formation occurred in the later GEs, which released less FA. This suggests that higher times of residence in the stomach increase FA oxidation. This study shows a higher formation of LOPs during digestion of FM using a whole meal approach. These results relate to its richness in PUFAs compared to BM. Despite higher LOPs formation, FM digests that reached duodenum still contain higher content of unoxidized PUFAs compared with BM and a desirable ω3/ω6 PUFAs ratio of ~0.43. LOPs formation in PUFA-rich meals could be reduced if those meals have a low caloric value, avoiding large times of residence in the stomach and consequently high levels of oxidation.

## Introduction

Meat and fish are usually included in meals as a source of protein, given their richness in high-quality proteins. Nonetheless, these muscle foods also present different fatty acid (FA) composition, which not only impacts the nutritional quality of the meals but also influences their stability under oxidative processes (1). The incorporation of these muscle foods in meals involves the combination of other food constituents and cooking methods, which may also play a role on oxidative reaction events (2). Regardless of the muscle food type and origin or external factors (e.g., storage and animal diet), lipid oxidation starts with slaughter of animals, continues along the food chain, and will not stop after the ingestion (3). The stomach has been recognized as a bioreactor of lipid peroxidation, given its acidic environment, presence of metallic ions, and dissolved oxygen (4). While fatty fish is expected to oxidize due to its higher content in polyunsaturated FA (PUFA), for red meat, the higher heme-Fe content may greatly contribute to several chemical oxidative processes causing lipid oxidation (5).

Dietary FAs are essential to the human body as a source of energy and as key factors to maintain the structural function of the cell membrane (6, 7). However, the human health benefits of FA are controversy because of their structural diversity in a wide range of foods, while saturated FAs (SFAs) have a recognized impact on inflammation being associated to the development of several inflammatory diseases, and the PUFA seems to have a protective effect toward inflammation (8). Thus, nutritional quality scores based on the FA profile of foods have been used to measure the impact on health based on prediction of atherogenic indexes (AIs) or thrombogenic indexes (TIs) and consequently potential of increasing risk of cardiovascular diseases (9).

Food sources high in PUFAs present two opposite effects in humans, namely, promotion of beneficial effects due to anti-inflammatory properties of PUFA and contribution to oxidative stress through exposure to hazardous substances as a result of PUFAs degradation through oxidation processes (10, 11). Actually, there are several mitigation strategies to minimize oxidation processes along the food chain (12–14), but the physiological oxidative processes that take place during gastrointestinal digestion will happen despite the presence of those antioxidant agents (15, 16). Floros et al. (17) recently stated that most of oxidation of PUFAs occurred in the gastric phase, reducing their availability to be absorbed, so it is of paramount importance to understand at what extent PUFAs remain unoxidized after gastric digestion, to retain the anti-inflammatory effects of PUFA-rich meals.

The oxidation of PUFAs is a dynamic process involving several steps and production of numerous types of oxidized products: first, the hydroperoxides are formed, which are highly unstable and rapidly decompose to reactive aldehydes [e.g., malondialdehyde (MDA) and 4-alkenals] (18). These later LOPs are harmful substances known to cause oxidative stress and to be involved in the development of several metabolic diseases (10, 18). Lipid oxidation of muscle foods from the mammal or aquatic origin during gastrointestinal digestion has already been assessed using *in vitro* static digestion models (1, 15, 19–22), but none of these studies considered the role of other food constituents (pro- or antioxidative behavior) toward oxidation or their influence on the caloric value of the meal, which affects the digestion time and, consequently, lipid oxidation.

The high reactivity of these intermediate LOPs and the quick conversion into tertiary oxidation products do not allow the quantification/measurement of the overall oxidation extent of a product. Therefore, this study focused on monitoring how lipid oxidation evolves during the gastric phase, as well as assessed the rate of passage of unoxidized FA and LOPs from the stomach to the duodenum compartment, bridging the gap of knowledge regarding FA oxidation in a whole meal approach. Moreover, the INFOGEST international network recently published a new semi-dynamic model of digestion that allows the adaptation of gastric digestion according to the caloric energy of meals, the simulation of gastric emptying's, the gradual secretion of digestive enzymes, and the pH acidification to better mimic physiological conditions (23), overcoming some issues faced when using *in vitro* static digestion models. This semi-dynamic digestion model has recently been applied in our research laboratory to study gastric lipolysis of milk and lipid oxidation (24). Thus, this model can be a suitable tool to monitor FA passage rate from the stomach to the duodenum during digestion of whole meals through gastric emptying's simulation, while assessing how lipid oxidation evolves during gastric digestion and where the greatest oxidation is expected to happen.

Therefore, this study used the semi-dynamic digestion model proposed by the INFOGEST to assess the passage of unoxidized FA from the stomach to the duodenum of two meal diets differing in animal protein source, namely, beef and mackerel, while monitoring how lipid oxidation evolves during the gastric phase.

## Materials and Methods

### Preparation of Meals

Beef, mackerel, potatoes, and sugary soft drinks were purchased from the retail food market in Porto, Portugal. Two types of meal diets were prepared in this study: (i) Beef meal (BM) diet containing roasted fatty beef (100 g), fried potatoes (100 g), and sugary soft drink (200 g) and (ii) fish meal (FM) diet composed by roasted mackerel (100 g), fried potatoes (100 g), and sugary soft drinks (200 g). The sugary soft drink used in each meal was the same, and it consisted of carbonated water, high-fructose corn syrup, caramel color, phosphoric acid, natural flavors, and caffeine. The potatoes were peeled, washed, and fried at 170°C with sunflower oil for 7 min. As for cooking of muscle foods, the beef was primarily sealed to avoid loss of nutrients, then covered in baking paper, and roasted in an oven at 200°C for 30 min, while the mackerel was roasted at 200°C for 15 min and also covered in baking paper. Both times of cooking were previously optimized to ensure an internal temperature of 77°C. After cooking and cooling, the heads, skins, bones, and tails of mackerel were removed. Further in the study, as a confirmatory experience of the results obtained, a new FM diet was prepared replacing the sugary soft drink by a 0% sugar drink (FM_NSD).

Subsequently, the different meals were prepared by weighting each food constituent and beverage, mixing using a food homogenizer, and packing in plastic vacuum containers for storage at −80°C until analysis. On the same day, the proximate composition of meal diet was determined as follows: moisture by oven-drying at 105°C following the AOAC 950.46 method (25); crude fat by Soxhlet following the AOAC 991.36 method (26); crude protein by Kjeldahl according to the AOAC 981.10 method (27); and the ashes by heating in a muffle furnace at 500°C according to the AOAC 920.153 method (28). Amino acid determination was performed by reversed-phase high-performance liquid chromatography (RP-HPLC) with fluorescence detection, with precolumn derivatization with 9-fluorenylmethyl chloroformate (FMOC) and O-phthaldialdehyde (OPA) as described by Pinho et al. (29).

### *In vitro* Semi-Dynamic Digestion of Meals

The simulation of human gastric digestion was performed following the standardized semi-dynamic protocol designed by Mulet-Cabero et al. (23). The fat, protein, and carbohydrate contents obtained from the proximate analysis of meal diets were used to calculate the energy value needed for simulating gastric digestion, that were of 121.4, 87.6, and 70.9 kcal/100 g of meal for BM, FM, and FM_NSD, respectively. The total amount of meal (400 g) was considered as real *in vivo* meal proportion to allow the scale down to an *in vitro* simulation using 25.0 g, using the protocol's supplementary information of Mulet-Cabero et al. (23).

#### Oral Digestion Step

For BM, the oral digestion was prepared by adding 5.524 ml of electrolyte-simulated salivary fluid (eSSF), 34.5 μl of 0.3M CaCl_2_(H_2_O)_2_, 0.345 ml of amylase solution (150 U/ml), and 1.001 ml of ultrapure water, with a final volume of oral phase of 31.9 ml. The oral digestion of FM and 4.210 ml of eSSF were mixed with 26.3 μl of 0.3 M CaCl_2_(H_2_O)_2_, 0.263 ml of amylase solution (150 U/ml), and 0.763 ml of ultrapure water, with a final volume of oral phase of 30.3 ml. As for FM_NSD meal, 3.91 ml of eSSF were mixed with 19.5 μl of 0.3 M CaCl_2_(H_2_O)_2_, 0.195 ml of amylase solution (150 U/ml), and 0.566 ml of ultrapure water, with a final volume of oral phase of 28.9 ml.

#### Gastric Digestion Step

The respective oral phase of each meal was transferred into a 70 ml Metrohm (ref.: 6.1418.150, Switzerland) glass v-form vessel thermostated at 37°C with a stirrer paddle at 15 rpm containing the basal simulated gastric fluid (10%, SGF) resembling the *in vivo* fasting conditions. The remaining 90% of SGF were added at a constant ratio by separated devices. For BM, the SGF was composed by 22.33 ml of electrolyte SGF (eSGF), 2.871 ml of enzyme solution [pepsin (4,000 U/ml) and RGE (120 U/ml)], 16.0 μ, of 0.3M CaCl_2_(H_2_O)_2_, 3.30 m, of 1.5 N HCl, and 3.07 m, of ultrapure water. In the meantime, the gastric digestion of FM was arranged by joining 21.18 ml of eSGF, 2.737 ml of enzyme solution [pepsin (4,000 U/ml) and RGE (120 U/ml)], 15.1 μl of 0.3M CaCl_2_(H_2_O)_2_, 3.30 ml of 1.5 N HCl, and 2.7 ml of ultrapure water. As for FM_NSD, 20.23 ml of eSGF were added to the bolus, along with 2.89 ml of enzyme solution [pepsin (4,000 U/ml) and RGE (120 U/ml)], 14.5 μl of 0.3M CaCl_2_(H_2_O)_2_, 1.2 ml of 2N HCl, and 4.57 ml of ultrapure water.

**Figure 1:**
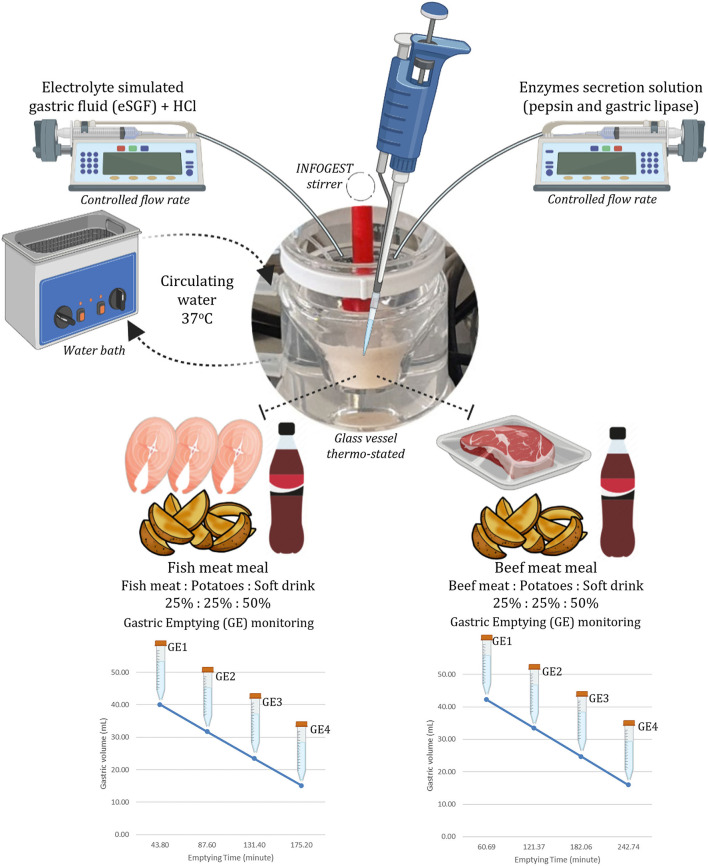
Scheme of *in vitro* gastric phase of the INFOGEST semi-dynamic digestion method.

The four GE points were sampled using a disposable plastic pipette by collecting 15.95 ml of each emptying every 60.9 min from the BM and every 43.8 min from FM. In the FM_NSD, aliquots of 14.45 ml were taken each 35.5 min. In each emptied aliquot, the pH was measured and raised to pH 8.0 to completely inactivate pepsin and gastric lipase (30). Two replicates for each meal and each emptying were performed. Figure 1 illustrates the structure of the digestion simulation experiment for the BM and FM. FM_NSD followed the same design. Table 1 provides the emptying times and volumes during the semi-dynamic gastric digestion of BM, FM, and FM_NSD.

**Table 1:** Overview of sampling times and volumes during the semi-dynamic *in vitro* gastric digestion in beef meal, fish meal, and fish meal without sugary drink diets.

**Type of meals**	**GE**	**Time GE (min)**	**Actual gastric volume (mL)**	**Secretion volume (mL)**
Beef meal diet		0.00	35.1	0.00
	GE 1	60.7	26.3	7.18
	GE 2	121.4	17.6	14.4
	GE 3	182.1	8.77	21.5
	GE 4	242.7	0.00	28.7
Fish meal diet		0.00	33.3	0.00
	GE 1	43.8	24.9	6.81
	GE 2	87.6	16.6	13.6
	GE 3	131.4	8.32	20.4
	GE 4	175.2	0.00	27.2
Fish meal without		0.00	31.8	0.00
sugary drink	GE 1	35.5	23.8	6.50
	GE 2	70.9	15.9	13.0
	GE 3	106.5	7.95	19.5
	GE 4	141.9	0.00	26.0

*The actual gastric volume relates to the volume of chyme in the vessel at each gastric emptying; the secretion volume refers to the volume of electrolytes and enzymes that were gradually added to the vessel at each gastric emptying*.

### Triacylglycerols and Total Fatty Acids Analysis

#### Fat Extraction

Lipid extraction followed the Bligh and Dyer method as defined by Pérez-Palacios et al. (31) with modification. Depending on GE, different sample volumes were taken (0.75 or 1.5 ml). For those using 0.75 ml of sample, the volume before extraction was adjusted up to 1.5 ml with 1% NaCl. Then, 50 μl of 10 mg/ml undecanoic acid solution (TAG_C11:0, internal standard) and antioxidants (50 μl of BHT and the tip of a spatula of ascorbic acid) were added. Then, 3 ml of methanol was added, and the solution was homogenized using a vortex. Furthermore, 1.5 ml of chloroform and 1.5 ml of 1% NaCl were added, and the solution was subsequently centrifuged (3,000 × *g* for 10 min). After the centrifugation, the lower phase (chloroform) was collected, while the upper phase was subjected to a second fat extraction by mixing with 550 μl of 3 M HCl and 3 ml of chloroform, centrifugation (3,000 × *g* for 10 min), and collection of the lower phase to join to previous chloroform extract. Water removal from chloroform extract was achieved by adding anhydrous Na_2_SO_4_ salts. Then, chloroform extract was evaporated to dryness under gentle nitrogen stream (Stuart®, Staffordshire, USA). Finally, the dried extract was dissolved with 1 ml of hexane and extracted fat divided into two parts, namely, (i) 500 μl were dried under nitrogen stream and dissolved in 10 ml of isooctane for conjugated diene and trienes analysis (refer to the “Determination of conjugated dienes and trienes” section), followed by glyceride analysis (refer to the “Glyceride analysis” section); while (ii) the other 500 μl were used for total FA analysis (refer to the “Fatty Acid Analysis” section).

#### Fatty Acid Analysis

For the FA analysis, 500 μl of fat extract obtained (as discussed in the “Fat Extraction” section) was mixed with 1 ml of 2% sulfuric acid in methanol, homogenized using a vortex, and left reacting overnight (15 h) at 50°C. After the reaction time, the samples were cooled at room temperature and mixed with 1 ml of neutralizing solution (aqueous 2.1% NaHCO_3_ and 6.9% K_2_CO_3_) and 1 ml of hexane. Furthermore, the samples were homogenized and centrifuged for 5 min at 100 × *g*, transferring the upper phase (hexane) to a 2-ml vial.

Fatty acids were determined by gas chromatography (Chrompack CP-9001 model, The Netherlands) with flame ionization detection (FID) as Cruz et al. (32). The chromatographic separation was achieved using a FAME CP-Select CB column (50 m × 0.25 mm × 0.2 μm; JW), with helium as carrier gas at 17 Psi, and a temperature gradient from 140 to 200°C, in a total of 40 min. The injection port was 250°C, with a 1:100 split ratio, and the detector was at 270°C. The FAs were recognized by comparison with commercial standards from Supelco (Sigma, USA) and Matreya (USA). Data were processed using the CP Maitre chromatography data system program (Chrompack International B. V., Middelburg, Netherlands, version 2.5). The FA analysis was only performed in BM and FM.

#### Glyceride Analysis

The fat extracts obtained (as discussed in the “Fat Extraction” section) were evaporated to dryness and dissolved in a 1:1 solution of methanol and tetrahydrofuran (THF). These extracts were eluted at a flow rate of 1 ml/min using THF as mobile phase through a high-performance size-exclusion chromatography (HPSEC) system (Jasco, Japan), equipped with a styrene-divinylbenzene copolymer R column (pore size 10 nm, 60 cm × 7 mm) and refractive index (RI) detection (Gilson, USA). Diverse FA, mono-, di-, and triglyceride standards were used as reference (Sigma, USA). The glyceride analysis was only performed in BM and FM.

### Lipid Oxidation Parameters Analysis

#### Determination of Malondialdehyde

Thiobarbituric reactive species (TBARS) were assessed as described by Sobral et al. (15). To 150 mg of sample (meal before digestion), 400 μl of digested (from each GE) or 400 μl of standard solution, 1 ml of 7.5% (w/v) trichloroacetic acid (TCA), and 40 μl of 4.5% (w/v) butylated hydroxytoluene (BHT) in ethanol were added to 1.5 ml microtubes and thoroughly homogenized to allow protein precipitation. The samples were then centrifuged (3,000 *g*, 5 min; Thermo Scientific, U.S.A), and 750 μl of supernatant was transferred to a new microtube. A second TCA precipitation step was repeated, and the supernatant of the second precipitation was combined with the supernatant of first precipitation. Then, 500 μl of supernatant was added to 500 μl of 40 mM thiobarbituric acid (TBA) reagent and left to react for 45 min at 90°C. The reaction was stopped by immersing the tubes on ice for 10 min. Then, the samples were centrifuged to remove any insoluble fragments, and 200 μl of each sample were transferred to wells in a microplate, and the absorbance values were measured at 532 nm in the spectrophotometer (SPECTROstar Omega, BMG Labtech, Germany). The TBARS content was calculated from a standard curve of 1,1,3,3-tetraethoxypropane (TEP) solution (ranging from 0.2 to 25.6 nmol) in 7.5% TCA. Results were expressed as total nmol of MDA equivalents. Four replicates were performed for each measurement.

#### Determination of Conjugated Dienes and Trienes

The conjugated diene, triene, and tetraene in lipid extract was measured according to Kim and LaBella's (33) method. The fat extract obtained (as discussed in the “Fat Extraction” section) was dissolved with 10 ml of isooctane and homogenized, and the absorbance of the solution was measured at 233 nm for dienes (*n2* series) and 268 and 278 nm for trienes, *n3* and *n4* series, respectively. The concentration of conjugated dienes and trienes was calculated using a molar extinction coefficient of 27,000 M^−1^ cm^−1^ (*n2* series), 43,400 M^−1^ cm^−1^ (*n3* series), and 33,500 M^−1^ cm^−1^ (*n4* series), and the results were expressed as nmol/mg of fat.

### Quality Indices of Meals

Lipid quality indices of meals, such as AI and TI, were calculated for BM and FM according to Fehily et al. (34) and Ulbricht and Southgate (35), and the peroxidisability index (PI) was determined according to Fernández et al. (36), based on the following equations:


$$\begin{array}{l} AI=\frac{\left[C12:0+4xC14:0+C16:0\;\right]}{\left[\sum MUFA+\;\sum \omega 6\;PUFA+\;\sum \omega 3PUFA\right]}\\ TI=\\ \frac{\left[C14:0+C16:0+C18:0\;\right]}{\left[0.5x\sum MUFA+\;0.5x\sum \omega 6\;PUFA+\;3x\sum \omega 3PUFA\;+\;\omega 3/\omega 6\;\right]}\\ PI=\left(0.025\;x\;monoenes\right)+\left(1\;x\;dienes\right)+\left(2\;x\;trienes\right)\\ +\left(4\;x\;tetraenes\right)+\left(6\;x\;pentaenes\right)+\left(8\;x\;hexaenes\right) \end{array}$$

### Statistical Analysis

The statistical analysis and graphs were performed using the GraphPad Prism 8.1.0 version for Windows. Variable's normality was assessed by the Shapiro-Wilk test. Statistical significance between amino acid and fat composition (SFA, MUFA, PUFA, trans-FA, ω-3, ω-6, ω-3/ω-6) and AI and TI of BM and FM were determined using the Holm-Sidak *t*-test method, with alpha = 0.05. In this study, independent batches were used to prepare the meal diets. For each meal, the digestion experiments were performed in duplicate. Then, the nutrients/oxidation analysis of each meal or gastric emptying aliquots was performed in triplicate for each parameter (*n* = 1 × 2 × 3 = 6 for each meal). Data are expressed as mean ± standard deviation.

## Results

### Nutritional Composition and Quality of Meals

**Figure 2:**
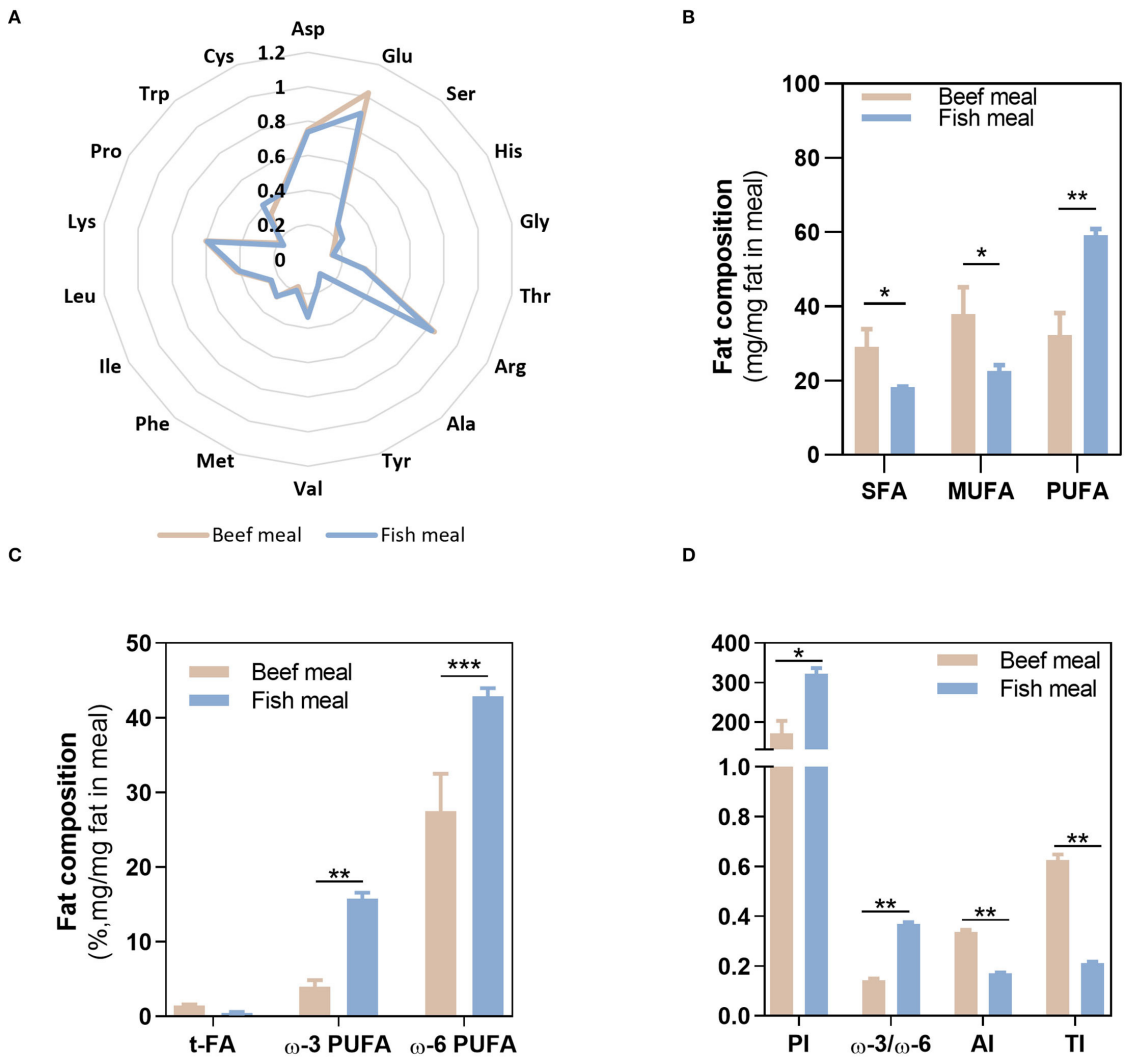
Fatty acids and amino acids profile. Composition of beef and fish meal diets concerning their amino acid profile **(A)**, fatty acid profile **(B,C)**, and meals quality indexes **(D)**. Asp, aspargine; Glu, glutamic acid; Ser, serine; His, histidine, Gly, glycine; Thr, threonine; Arg, arginine; Ala, alanine; Tyr, tyrosine; Val, valine; Met methionine; Phe, phenylalanine; Ile, isoleucine; Leu, leucine; Lys, lysine; Pro, proline; Trp, typtophan; Cys, cysteine; SFAs, saturated fatty acids; MUFA, monounsaturated fatty acids; PUFA, polyunsaturated fatty acids; t-FA, trans-fatty acids; PI, peroxidisability index; AI, atherogenic index; TI, thrombogenic index. *p < 0.05, **p < 0.01, ***p < 0.001.

The nutritional composition of BM and FM differed on the total protein and fat contents, with BM having higher amounts (10.4 ± 0.70% protein and 2.77 ± 0.12% fat) compared with FM (6.85 ± 0.35% protein and 1.24 ± 0.02% fat). Despite the highest protein content in BM, the amino acids profile was similar between both meals, as shown in the radar graph (Figure 2A). In contrast, FA profile significantly differed between BM and FM (Figures 2B,C). As expected, BM had the highest content of SFA and MUFA (together represented more than 65% of the fat in this meal), while FM was significantly richer (*p* < 0.01) in PUFAs (~60% of the fat), both ω-3 (15.8 ± 0.75%; *p* < 0.01) and ω-6 (42.9 ± 1.09%; *p* < 0.001) PUFAs. As for trans-FA, no statistical differences were observed between meals.

Given the distinct FA profile of BM and FM meals, different meal quality indexes values—PI, AI, and TI indexes—were observed. The highest value of PI (322 ± 14) was obtained in FM as a result of its richness in PUFAs and, therefore, higher susceptibility to oxidation. On the contrary, BM shows the highest scores for AI (0.34 ± 0.01) and TI (0.63 ± 0.02), justified by its high SFA content. Thus, regarding those quality indexes, FM reveals the highest ω-3/ω-6 ratio (~0.4), lowest AI, TI, but its high PUFAs content along with the oxidative environment of the stomach may favor lipid peroxidation, contributing to the loss of nutritional value of FM.

### Semi-Dynamic Fat Release From Stomach to Duodenum

**Figure 3:**
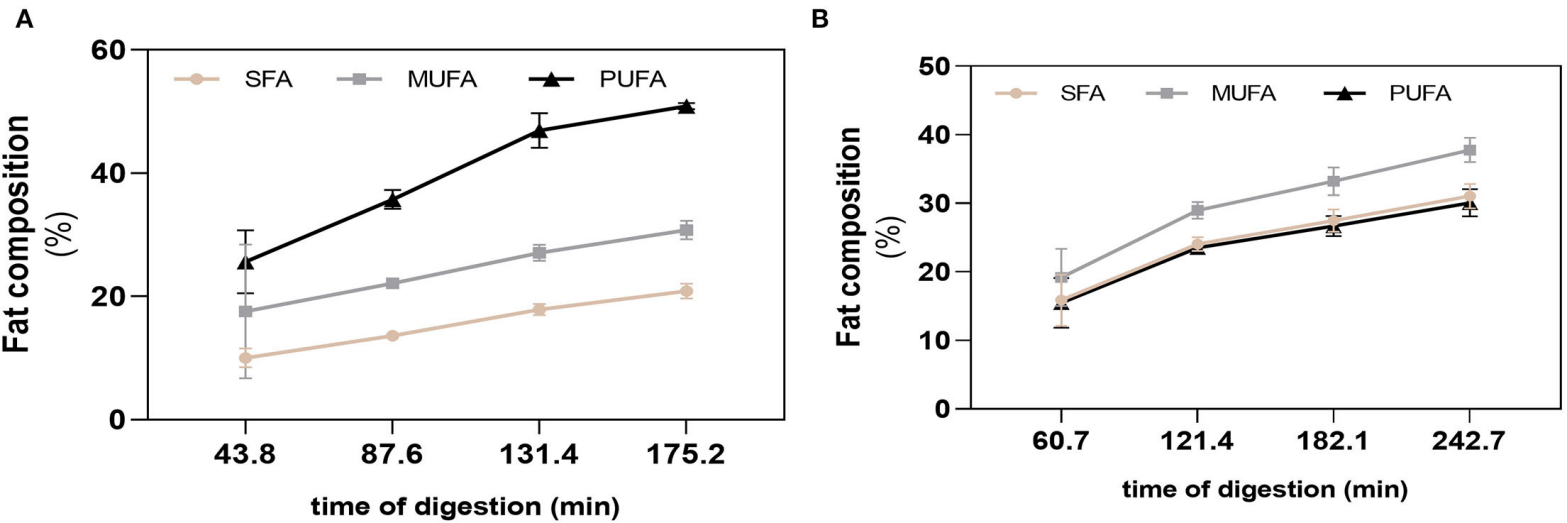
Fatty acid cumulative release during digestion. SFA, MUFA, and PUFA release in each gastric emptying for fish **(A)** and beef **(B)** meal diets. Data are presented as the average ± SD of eight replicates (*n* = 8). SFA, saturated fatty acids; MUFA, monounsaturated fatty acids; PUFA, polyunsaturated fatty acids.

The differences on nutritional composition of BM and FM influenced the total gastric digestion time and respective emptying times, with BM having a larger gastric digestion time of 242.74 min, while the gastric digestion time of FM was of 175.20 min. The four GE were simulated at each 60.9 (BM) and 43.8 min (FM). The fat of each GE was analyzed to follow the passage of unoxidized FA from the stomach to the duodenum. The action of gastric lipase during gastric digestion was confirmed by the decrease of triacylglycerides (TGs) and the consequent increase of free FA (FFA), observed in both meals right after the first GE. After 43.8 min of digestion of FM, the first fat release to the duodenum was mainly composed by PUFAs, followed by MUFAs and SFA, respectively (Figure 3A). An increase of 10.1% (PUFA), 4.5% (MUFA), and 3.6% (SFA) was verified in the GE2 and GE3, whereas GE4 showed the lowest FA release with percentages of SFA, MUFA, and PUFAs ranging from 3.0 to 3.9%. Overall, FM had a cumulative fat composition during digestion of 50.8% (PUFA), 30.8% (MUFA), and 20.9% (SFA), which shows a 10% loss of PUFA (*p* < 0.0001) compared with PUFA profile observed before digestion (Figure 2B), probably due to oxidation. In the case of BM (Figure 3B), within each GE, the different types of FA were released at similar percentages exhibiting a cumulative release of FA of 37.8% (MUFA), 31.0% (SFA), and 30.1% (PUFA), which did not differ from the initial fat composition observed before digestion.

**Figure 4:**
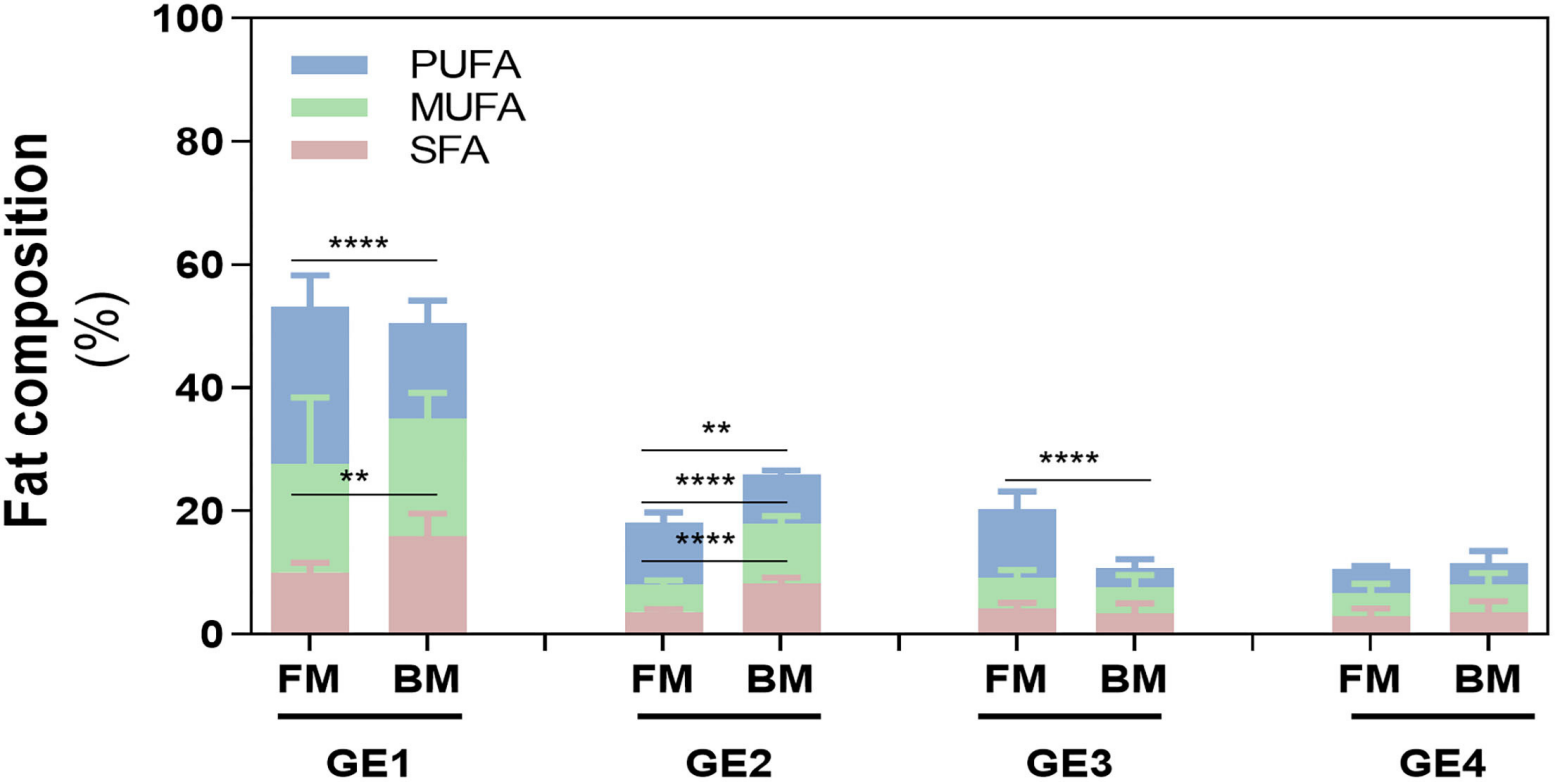
Fatty acid (FA) composition during digestion. Release of FA in each gastric emptying (GE) of beef and fish meal diets. Data are presented as the average ± SD of eight replicates (*n* = 8). Parametric (*t*-test) statistical tests were applied to compare FA release between beef or fish meals, and statistical differences are indicated as **p < 0.01 and ****p < 0.0001. GE1 to GE4 mean gastric emptying 1 to gastric emptying 4. FM, fish meal; BM, beef meal; SFA, saturated fatty acids; MUFA, monounsaturated fatty acid; PUFA, polyunsaturated fatty acid.

Particularly, in GE1, even though the total amount of FA released was similar in both meals (~50%), SFA and PUFA content differed between meals (*p* < 0.01 and *p* < 0.0001), which was not observed in the release of MUFAs (Figure 4). Fat release was inversely proportional with time of gastric digestion: GE1>GE2>GE3>GE4 for BM and GE1>GE2=GE3>GE4 for FM. This is explained because GE1 represents the first chyme passage to the duodenum, and therefore, it transfers the highest FA content to the duodenum with the lowest dilution promoted by gastric fluids (Table 1). Interestingly, GE2 and GE3 show an opposite FA content between meals: FM had a lower contribution in GE2 (~18%) related to BM (26%), but in GE3, a higher FA release was verified in FM (~20%) rather than BM (10%), whose 10% difference is related to PUFAs content. FA release in GE4 did not statically differ between meals.

Focusing on PUFAs release, ω-6 PUFA of FM and BM showed the greatest cumulative release during digestion, followed by ω-3 PUFA of FM (Table 2). The contents of both ω-3 and ω-6 PUFAs differed between meals in GE1, with FM showing higher amounts of PUFAs. The same trend (ω-6>ω-3) was verified in the other GEs, as confirmed by the ω-3/ω-6 ratio.

**Table 2:** Cumulative release (%) of ω-3 and ω-6 PUFA along gastric emptying (GE) for beef meal (BM) and fish meal (FM) diets.

	**Cumulative release**			
	**ω-3 PUFA**		**ω-6 PUFA**		**ω-3/ω-6**	
	**BM**	**FM**	**BM**	**FM**	**BM**	**FM**
GE1	1.90 ± 0.48	7.72 ± 2.30	13.1 ± 3.03	17.6 ± 2.82	0.145 ± 0.004	0.429 ± 0.067
GE2	2.88 ± 0.06	10.6 ± 0.39	19.8 ± 0.51	24.7 ± 1.13	0.145 ± 0.001	0.430 ± 0.009
GE3	3.28 ± 0.19	14.1 ± 0.60	22.5 ± 1.22	32.3 ± 2.19	0.146 ± 0.001	0.438 ± 0.012
GE4	3.69 ± 0.22	14.9 ± 0.48	25.3 ± 1.70	35.3 ± 0.17	0.146 ± 0.001	0.425 ± 0.014

*Data are expressed as average ± SD of eight replicates*.

When comparing with the initial values of meal before digestion, the ω-3 PUFAs content of FM was not affected by gastric digestion, only a slight decrease from 15.8 ± 0.75 to 14.9 ± 0.48% was observed, while a reduction of 8% (from 42.9 ± 1.09 to 35.3 ± 0.17) was observed in ω-6 PUFAs content (*p* < 0.0001). The degree of oxidation of BM was not significant to decrease the PUFA content, exhibiting similar values as the ones measured in BM before digestion (3.97 ± 0.88 and 27.5 ± 4.95 for ω-3 PUFA and ω-6 PUFAs, respectively). These results could suggest a loss of nutritional value of FM through oxidation of essential ω-6 PUFAs and formation of hazardous substances. However, despite the loss of PUFAs during gastric digestion, FM continues to exhibit a greater PUFA content in comparison with BM: total PUFAs, ω-3 and ω-6 PUFAs, and ω-3/ω-6 ratio.

### Fat Oxidation During Semi-Dynamic Digestion

**Figure 5:**
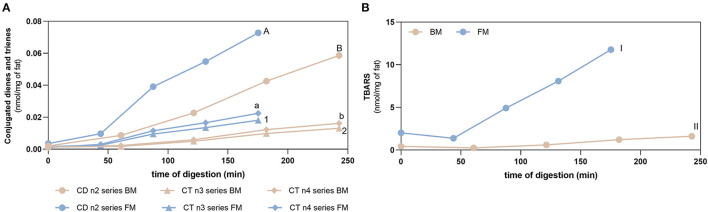
Fat oxidation. Formation of conjugated dienes (CD, n2 series) and conjugated trienes (CT, n3, and n4 series) **(A)** and TBARS **(B)** during gastric digestion of beef and fish meal diets. Data are presented as the average ± SD of four replicates (*n* = 4). Parametric (*t*-test) statistical tests were applied to compare FA oxidation at the end of gastric digestion between beef or fish meal diets, and statistical differences (*p* < 0.0001) are indicated using different letters or numbers (uppercase letters for CD n2 series, lowercase letters for CT n4 series, Arabic numbers for CT n3 series, and Roman numerals for TBARS values).

Figure 5 shows the evolution of lipid oxidation during gastric digestion of BM and FM measured by the formation of conjugated dienes/trienes (CD/CT) as primary oxidation product markers and TBARS (secondary oxidation products). Prior to digestion, both meals had already an initial degree of oxidation: (i) BM and FM had values of CD below 0.002 and 0.004 nmol/mg of fat, respectively; (ii) CT (n3 and n4 series) values below 0.001 and 0.002 nmol/mg of fat for BM and FM, respectively; and (iii) TBARS values of 0.43 ± 0.01 and 2.02 ± 0.11 nmol/mg of fat for BM and FM, respectively. Initial levels of oxidation of meals were expected as oxidative reactions start right after slaughter of animal and continue along transport, storage, and thermal treatment of foods.

**Figure 6:**
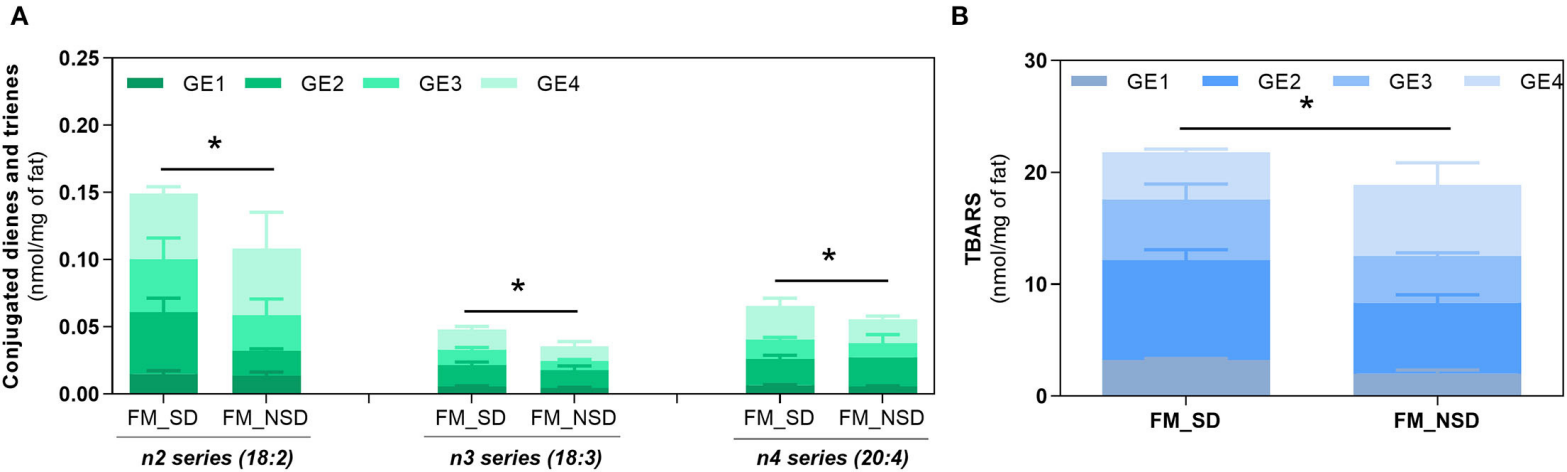
Fat oxidation of fish meal containing sugary drink (FM_SD) vs. fish meal without sugary drink (FM_NSD). Formation of conjugated dienes (CD, n2 series) and conjugated trienes (CT, n3, and n4 series) **(A)** and TBARS **(B)** during gastric digestion of FM and FM_NSD. Data are presented as the average ± SD of four replicates (*n* = 4). Parametric (*t*-test) statistical tests were applied to compare FA oxidation at the end of gastric digestion between FM and FM_NSD meal diets. *Indicate statistical differences (*p* < 0.05) between meals.

Gastric digestion triggered the formation of CD and CT, as well as TBARS confirming the high PI index previously calculated for FM and the susceptibility of PUFA-rich meals to oxidize during digestion. When comparing with BM, FM showed the highest formation (*p* < 0.0001) of all these LOPs. The lowest formation of LOPs was observed in GE1. The following GEs showed a similar lipid oxidation extent, except for CD and CT of FM, where GE2 showed a higher contribution. Regarding the TBARS analysis, a massive increase was observed for FM in comparison with the initial content before digestion and the maximum amount observed in BM after gastric digestion (*p* < 0.0001). In addition, it seems that the formation of LOPs does not follow the trend of FA release from the stomach to the duodenum: higher levels of CD, CT, and TBARS are observed in the later GEs that contribute with the lowest release of FA to the duodenum. These are interesting results suggesting that the first emptying, that represents 50% of FA release, is less prone to oxidation and that the higher the time of residence in the stomach the higher the oxidation of PUFAs. A new set of experiments was performed to confirm those results undertaking digestion studies of two different fish diet meal, one as is (with the inclusion of the sugary drink), and another diet replacing the sugary drink by a 0% sugar drink. This replacement reduced the carbohydrate content of the meal and consequently the caloric energy value, which reduced the time of residence in stomach. Figure 6 shows that the FM diet without sugary soft drink (FM_NSD) exhibits a statistically (*p* < 0.05) lower formation of LOPs compared with FM diet with sugary soft drink (FM_SD).

## Discussion

In this study, the FA profile and oxidative stability of two meals differing in animal source of protein (i.e., beef or fish) was monitored during gastric digestion, knowing that the animal origin has a major impact on FA profile. The FA profile of FM before digestion (ω3 < SFA < MUFA < ω6) agrees with previously published data of mackerel samples combined with sunflower oil (ω-6 PUFA-rich oil), while the FA profile of BM (ω3 < ω6=SFA < MUFA) differed from those of beef samples combined with sunflower oil in the same report (37). Although ω-6 PUFAs like the linoleic acid (C18:2) are essential FA to our health, an excessive amount of ω-6 PUFAs in meals increases ω6/ω3 PUFAs ratios and may favor the development of several diseases, including cardiovascular disease, cancer, and inflammatory diseases (38). Despite its high ω6 PUFAs content, FM also has a high content of ω3 PUFAs known to exert suppressive effects of those diseases, which may counterbalance the effects of ω6 PUFAs (38). In fact, FM offers a desirable ω3/ω6 ratio of 0.37 ± 0.01 in comparison with BM (0.14 ± 0.006), as well as better nutritional quality indexes: AI and TI that agree with the recommended values in diet (AI < 1.0 and TI < 0.5) regarding human health safety (39). In 2018, Thomsen et al. (40) investigated the risk-benefit of replacing red meat with fish in Danish diet, reporting an overall beneficial effect on the substitution, but emphasizing some constraints regarding fish contamination (e.g., dioxins and methyl mercury). Thus, although fatty fish offers numerous health benefits, risk-benefit assessments of fish intake are needed and should consider the unstable nature of PUFAs.

After the ingestion of a meal, the gastric phase of digestion is of paramount importance allowing the disintegration of food into nutrients at a molecular level. This is achieved due to the acidic environment along with pepsin activity that denatures the protein structure. By doing that, lipid-protein complexes are broken down and together with gastric lipase a partial fat disintegration takes place in the stomach (41). Herein, rabbit gastric lipase was used for simulating human gastric lipolysis, which have proved to have a similar specificity of human gastric lipase (42). Along with gastric digestion, the chyme kept the same FA profile measured in meals, i.e., the chyme of FM contributed with higher contents of PUFAs in all GEs, while digests of BM were mostly composed of SFAs and MUFAs. Of those 50% of FA released in GE1 (Figure 4), some were already released in the form of FFA, given the larger time of gastric emptying of 43.8 or 60.7 min that allowed the hydrolysis of TG. Moreover, the activity of gastric lipase is pH-dependent with a spectrum of optimum pH ranging from 3.0 to 5.0 (43), which agrees with the pH of the first GEs in the present study, where most of hydrolysis occurred.

The food matrix/meal that is being digested has strong influence on the pro-oxidant environment of the stomach (2). FM presented a higher PI (322 ± 14) compared with 170.8 ± 31.2 of BM, which agrees with previous studies pointing fish samples as highly susceptible for oxidation (44–47). FM exhibited the highest formation of primary and secondary lipid oxidation products along gastric digestion: CD, CT, and TBARS. In addition, it was observed that the higher the time of residence of FA in the stomach, the higher their susceptibility to oxidize. The massive increase of TBARS reflects the formation of dietary aldehydes deriving from lipid peroxidation as MDA and perhaps 4-alkenals [e.g., 4-hydroxy-nonenal (HNE) and 4-hydroxy-hexenal (HHE)]. According to Maestre et al. (48), the oxidation products formed during gastric digestion are bioaccessible in jejunal and ileal compartments. Moreover, the same authors reported a 10% intestinal uptake of oxidized PUFAs using Caco-2 cells as the model. The oxidized lipids intake has been suggested to contribute to hypertension and atherosclerosis *via* inflammatory pathway (49). Alike lipids, proteins are also recognized players on oxidation of muscle foods (50); however, as the same amino acid profile was observed (Figure 2B), it could be presumed that most differences on oxidation between both meals are more likely due to lipid oxidation rather than protein oxidation. For this reason, the evolution of protein oxidation was not explored in this study. A study reported by Van Hecke et al. (1) showed that among several mammals, poultry, and fish muscle foods, BM had the lowest amount of LOPs in their digests due to their low content of oxidizable PUFAs, whereas mackerel was the fish species with the highest LOPs formation, including HHE and HNE. Floros et al. (17) reported a strong correlation between the oxidation rate of each FA and its initial concentration. Moreover, Dasilva et al. (51) observed different resistance to oxidation depending on PUFAs type present in meal, for example, a significant increase in CD happened when the ω3 docosahexaenoic acid (DHA) was present at higher levels, whereas diets enriched with linoleic acid were the most resistant to gastric oxidation. The same authors also stated that the higher the degree of unsaturation and number of carbons of the PUFA, the lower its stability under pro-oxidant conditions of the stomach (51). These data corroborate the highest oxidation observed in FM. These reactive aldehydes are cytotoxic and genotoxic compounds (52), and their presence in the diet and continuous formation during digestion could have deleterious effects on the digestive tract of humans and contribute to oxidative stress compromising the homeostasis of body. For example, the oxidation of PUFAs during digestion of turkey meat was related to increased circulating levels of oxidized low-density lipoprotein (ox-LDL) in humans (53) (ox-LDL is considered a proatherosclerotic factor). Likewise, the administration of a red meat diet with sunflower oil increased the levels of ω-6 PUFA oxidation products and ox-LDL in plasma of rats and also caused endothelial dysfunction and atherosclerosis compared with rats without added sunflower oil (54). However, LOPs formation seem not to follow the trend observed for FA release as higher or equal oxidation degree (CD, CT, and TBARS) was observed in the later GEs, the ones releasing less FA. Thus, the first FA release to the duodenum (GE1) represents the FA portion that likely contains the highest content of unoxidized FA. This suggests that higher times of residence in the stomach increase FA oxidation. The digestion of an FM without sugary drink (FM_NSD, Figure 6), and therefore, lower caloric energy, confirmed a lower LOPs formation after gastric digestion. In this sense, the preparation of meals containing foods rich in PUFAs (e.g., fish) should have a low caloric energy value to have a short gastric digestion time and avoid large times of residence in the stomach.

Even though FM exhibits the highest formation of LOPs during gastric digestion, its digests still offer the highest PUFAs content compared with BM (Figure 3), the most desirable ω3/ω6 PUFAs ratio of ~0.4, and a similar ω-3 PUFA content than the one measured before digestion (Figure 2 and Table 2). The later result was not expected, since the literature reports a high susceptibility of ω3 PUFAs to oxidation (37, 55). In addition, Van Hecke et al. (1) recently hypothesized that ω3 PUFA could possibly protect ω6 PUFA from oxidation by mechanisms of “sacrificial oxidation,” but this hypothesis was not confirmed in a later study published by the same authors (37). Moreover, the above-mentioned studies did not consider a whole meal approach, studying only fish/meat muscles cooked with addition or not of vegetables oils. Thus, further investigation should be made for in-depth understanding the interplay of the oxidation of ω-6 and ω-3 PUFAs in meals during digestion, considering the influence of several food components present in a meal dish as a more realistic approach.

In sum, when preparing a whole meal or performing dietary recommendations based on dietary fat intakes, the degree of unsaturation of FA present in foods should be considered, given the unstable nature of these types of FA. In this study, it was observed that despite the meal diet composed by fish (FM) presenting the best nutritional quality indexes before and after digestion, it also showed the highest formation of hazardous substances during gastric digestion, which can have deleterious effects on human health. According to this study results, the formation of those compounds can be reduced if the ingestion of foods rich in PUFAs is included in meals that have a low caloric content in order to avoid large times of residence in the stomach and therefore high levels of oxidation.

## Data Availability Statement

The raw data supporting the conclusions of this article will be made available by the authors, without undue reservation.

## Author Contributions

MS and IF: conceptualization, validation, formal analysis, data curation, and supervision. IH, MS, SC, SP, and MF: methodology and investigation. IH and MS: writing the original draft preparation. IF, SC, SP, MF, and JS: writing, reviewing, and editing the manuscript. IF: resources and funding acquisition. All authors have read and agreed to the published version of the manuscript.

## Funding

This study was financed by FEDER—European Regional Development Fund funds through the COMPETE 2020—Operational Program for Competitiveness and Internationalization (POCI) and by Portuguese funds through FCT—Fundação para a Ciência e a Tecnologia in the framework of the project POCI-01-0145-FEDER-030322—PTDC/SAU-NUT/30322/2017.

## Conflict of Interest

The authors declare that the research was conducted in the absence of any commercial or financial relationships that could be construed as a potential conflict of interest.

## Publisher's Note

All claims expressed in this article are solely those of the authors and do not necessarily represent those of their affiliated organizations, or those of the publisher, the editors and the reviewers. Any product that may be evaluated in this article, or claim that may be made by its manufacturer, is not guaranteed or endorsed by the publisher.
